# Media Usage and Adherence to the Mediterranean Diet in Children

**DOI:** 10.3390/nu16203481

**Published:** 2024-10-14

**Authors:** Alessandra Buja, Andrea Miatton, Anna Zanovello, Filippo Brocadello, Tatjana Baldovin, Marian Nur Muhiddin, Ilaria Spreghini

**Affiliations:** Department of Cardiac, Thoracic, Vascular Sciences, and Public Health, University of Padua, 35131 Padua, Italyanna.zanovello.1@studenti.unipd.it (A.Z.);

**Keywords:** Mediterranean diet, healthy eating, children, screen time

## Abstract

Background: Increased screen time in children is significantly associated with lower adherence to the Mediterranean diet (MD). The purpose of this study was to explore the association between different types of media use and the adherence to the MD in children. Methods: The study sample included 332 children aged 10–11 years attending the fifth year of primary school in Veneto, north-east Italy. The children’s mothers were surveyed on their children’s adherence to the MD, using the KidMed questionnaire, and on the time of media use, using the Media Activity Form-Parent (MAF-P). A multivariable backward stepwise linear regression was applied, adjusting the association for other potential confounding factors. Results: Children’s primary digital activities were watching TV or streaming platforms (5.0 h/week) and video content (2.5 h/week), followed by digital games (2.3 h/week). Lower adherence to the Mediterranean diet was associated with digital games (regression coefficient −0.65, SE 0.29, *p* = 0.026) and time spent watching TV or streaming platforms (regression coefficient −0.60, SE 0.29, *p* = 0.04). Conclusion: Interventions to promote healthier diets justify an approach that includes media education. Providing parents with recommendations on children’s use of digital media devices can empower them to improve their children’s well-being.

## 1. Introduction

In recent years, the growing presence of digital media has revolutionized several aspects of daily life, particularly for children and adolescents [1,2]. The proliferation of smartphones, tablets, and other digital devices has facilitated unprecedented access to a vast array of content, including video games, social media, and streaming services [3,4]. This surge in digital consumption has sparked concerns among researchers, educators, and health professionals regarding its potential impacts on children’s health and well-being [5,6].

One area of significant concern is the relationship between screen time and dietary habits [7]. Children who spend more time engaged in digital activities often exhibit poorer eating habits, marked by higher energy intake and frequent consumption of processed foods that are high in unhealthy sugars and fats but low in essential nutrients [8,9]. This association can be attributed to several factors, including increased exposure to food advertisements promoting unhealthy foods [10] and the development of irregular meal patterns, such as skipping breakfast and having late-night snacks [11,12]. In addition, the sedentary nature of digital engagement contributes to the development of unhealthy habits, which may continue into adulthood [13].

According to the Dietary Guidelines for Americans 2020–2025, by the age of two, children’s dietary recommendations largely align with those of adults [14]. However, the transition between late childhood and adolescence is a critical period for establishing the dietary patterns that will support the increased nutritional needs of puberty. During this stage, children gain more independence in their food choices as the number of meals eaten away from home increases [15]. This shift often leads to a preference for foods high in fat and added sugars [16]. Consequently, dietary patterns in late childhood show deficiencies in the intake of fruit, vegetables, and dairy products, while the recommended amounts of added sugars (10% of total energy), saturated fat (10% of total energy), and sodium (1.8 g) are often exceeded [14].

The Mediterranean diet (MD) is well known for its health benefits, including a reduced risk of metabolic diseases, immunological deficiencies, and neurological disorders [17,18,19,20]. It is characterized by a high consumption of fruits, vegetables, whole grains, and healthy fats, as well as a low intake of processed foods and sugars, and it is a benchmark for evaluating healthy eating patterns [21,22]. The Mediterranean diet also helps overall well-being by encouraging shared family time and a positive approach to mealtimes [23,24]. Adherence to this diet is especially important during childhood, a critical period for growth and development [25,26]. Emerging evidence suggests that excessive screen time may be inversely associated with adherence to the dietary recommendations of the Mediterranean diet in children [27]. The results of several studies are consistent with the fact that increased screen time reduces the intake of fruits, vegetables, and whole grains in both females and males, as well as increasing the consumption of energy-dense foods, such as energy-dense drinks and snacks, which results in increased energy intake from fat [8,28,29].

The present study aims to explore the association between consumption of a variety of digital contents and adherence to the Mediterranean diet in children. Understanding the role of emerging trends in children’s use of digital media is crucial for developing effective public health strategies and educational programs to promote healthier lifestyles.

## 2. Materials and Methods

### 2.1. Study Design

The present cross-sectional study derives from a survey administered in March–May 2023 as part of an educational intervention called “Le Buone Abitudini” [Healthy Habits], running since the academic year 2018/2019 at primary schools in the province of Padua (north-east Italy) [30]. This project promotes the adoption of a varied, healthy, and nutritionally balanced diet during childhood. Further details about the intervention are available elsewhere [31].

Of the 69 state primary schools in the province, 38 were invited to take part and 14 agreed, participating with at least one class. Participation in this study was offered to all children attending one of the classes selected for the intervention. Children whose parents did not provide signed informed consent were excluded from this study. No other exclusion or inclusion criteria were applied.

Our study sample included a total of 332 fifth-grade children (10–11 years old) attending 22 different classes. The children’s mothers were asked to answer a self-administered ad hoc online questionnaire. This study was conducted on anonymized data.

### 2.2. Materials

The questionnaire contained 34 multiple-choice items relating to both the children and their parents and touched on a number of factors considered to be potentially associated with the risk of poor adherence to the MD, i.e., social sphere and demographics, family setting, lifestyles (apart from nutrition), and behavioral traits.

The lifestyle factors considered in this study include the variable ”hours of sleep”, which encompassed both night-time sleep and daytime naps, the amount of time dedicated to homework (none, less than 1 h, or 1 h or more), and whether the children participated in sports or other out-of-school activities.

The “adherence to the MD” variable was derived from the Italian version of the KidMed Test [32]. Its development took into consideration aspects sustaining Mediterranean dietary patterns and also those undermining it. Previous studies showed that the KidMed questionnaire is a reliable instrument for assessing adherence to the Mediterranean diet, with a Cronbach’s alpha coefficient of 0.72 according to a Brazilian study [33,34]. The KidMed index ranges from 0 to 12 based on a 16-question test. Questions were assigned a value of −1 if they had a negative connotation towards adherence to the MD and +1 if they had a positive connotation. Children with scores of 3 or less were classified as having poor adherence to the MD, while those with scores of 4 or more were classified as having moderate-to-good adherence.

Children’s behavioral traits were measured on the basis of their mothers’ reports, using the Italian version of the Strengths and Difficulties Questionnaire (SDQ) [35]. The internal consistency of the SDQ was evaluated in a previous study that demonstrated a Cronbach’s alpha value of 0.70 in a large sample of Italian children [36]. The SDQ uses a 3-point Likert scale (from 0 = “not true” to 2 = “very true”) and consists of five subscales investigating emotional symptoms, hyperactivity/inattention, peer relationship problems, conduct problems, and prosocial behavior. Risk tertiles are identified for each behavioral trait: the first tertile identifies the children who exhibit a certain behavioral trait less, the second and third those who exhibit it more.

The use of media (“media” refers to all devices used to access the network such as smartphones, tablets, computers, video games, etc.) and its effect on the children were investigated in a dedicated section of the questionnaires using the Italian translation of the Media Activity Form-Parent (MAF-P) (T.M. Achenbach; reproduced under License 2606-02-02-23). Thirteen questions were used to gather information about parents’ perceptions of their children’s habits. A 3-point Likert scale (ranging from 0 = “not true” to 2 = “very true”) was used, and an individual’s final score was calculated by summing up the scores of each question, calculating an overall media use score. The survey also requested information on the amount of time spent on media-related activities during both weekdays and weekends. The total time spent on each activity per week was calculated by adding up the values for each day.

To categorize the variables, the tertiles of the distributions for media use score and time spent on online and digital activities were identified. The first and second tertiles were grouped together, while the third tertile was considered separately.

The second part of the questionnaire covered socio-economic aspects referring to parents and family environments, including the parent’s citizenship (Italian or other); the parent’s education (middle school or less, high school diploma, university degree); the parent’s marital status (married/cohabiting or unmarried/separated/divorced/widow); the parent’s need for help in understanding material provided by the doctor or pharmacist (never/rarely or occasionally/frequently/always); and the family’s disposable income. This last item was measured with the question “How do you make ends meet with your finances?” (very easily, quite easily or with some/great difficulty).

### 2.3. Statistical Analyses

Categorical variables were described by their absolute frequency and percentage. Quantitative variables were described with the median and interquartile range (IQR) because the Shapiro–Wilk normality test was rejected. Violin plots or boxplots were used for the representation of score-based variables and durations.

Differences in frequency distribution in the Mediterranean adherence group were assessed using the chi-square test or the Fisher test. The latter was only used when there were fewer than five absolute frequencies in the contingency tables. The Mann–Whitney test was used when one of the two variables was continuous or discrete.

Finally, a multivariable stepwise backward linear regression of the KidMed score was performed with a backward selection of the independent variables including all the lifestyle factors and the socio-economic factors referring to parents and family environments.

The missing values were treated in different ways according to the variable. When possible, the KidMed category (poor, medium, high) was deduced when a single answer of the KidMed questionnaire was missing. When there was more than one missing value, the subject was excluded from the analysis. The same approach was used for the parental style. For missing values in other variables, complete case analysis was used.

Results were deemed statistically significant when *p* < 0.05. The R software (ver. 14) was used for all the statistical analyses.

## 3. Results

A summary of sample characteristics is presented in Table 1. The sample included 332 children aged 10 to 11 years (mean 10.25, SD 0.45). Most mothers reported that their children had a medium or high adherence to the MD (86.2% of the sample). Based on the anthropometric measures provided, 66.6% of the children were regular weight, 18.4% were overweight, 10.7% were obese, and 4.3% were underweight.

**Figure 1:**
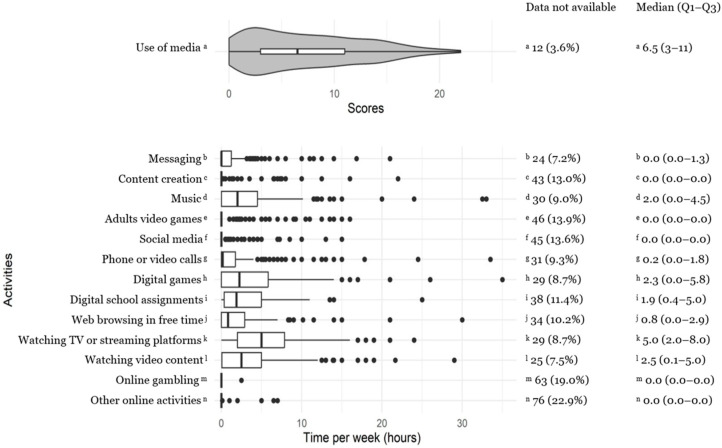
The sample’s distribution of scores for media usage and time spent on online and digital activities.

Figure 1 reports the sample’s distributions relative to media usage score and time spent on online and digital activities. Children’s main digital activities involved watching TV or streaming platforms (average 5.0 h per week, IQR 2.0–8.0) and watching video content (average 2.5 h, IQR 0.1–5.0), followed by digital games (average 2.3 h, IQR 0.0–5.8).

According to the bivariate analysis (Table 2), the media use score (median 6.5, IQR 3–11) was not associated with significant changes in children’s habits relative to the Mediterranean diet (*p* = 0.292). By contrast, children who spent more time playing digital games (*p* = 0.001) or watching TV and using streaming platforms (*p* = 0.002) were significantly associated with lower adherence to the MD.

The multivariable linear regression of the KidMed score (Table 3) showed similar associations with regard to digital games (regression coefficient −0.65, SE 0.29, *p* = 0.026) and time spent watching TV or using streaming platforms (regression coefficient −0.60, SE 0.29, *p* = 0.04).

## 4. Discussion

This study explored the association between the consumption of various forms of popular media among a sample of 10- to 11-year-old school-going children and adherence to the Mediterranean diet. The results indicated that playing electronic games and watching TV or streaming content are significantly associated with a lower adherence to the Mediterranean diet.

In recent years, the relationship between children’s eating habits and digital media has attracted interest in public health research. Most studies found that screen time was negatively associated with diet quality [8,9] and adherence to specific dietary patterns, such as the Mediterranean diet [37,38]. As van Sluijs and colleagues noted in their review of adolescent behavior [39], however, classic indicators such as television viewing and computer use are no longer adequate in representing the multitude of ways in which adolescents consume media. According to a recent report by Ofcom (the UK regulator for communications services) [4], although TV is still the most popular media device, with a household penetration rate of 97%, children now have access to a wide variety of connected devices, including tablets, laptops, video game consoles, smart speakers, and desktop computers.

In this study, the main form of digital entertainment for the sample of 10–11-year-old children was watching TV, which accounted for a large part of the time spent on the screen (5 h/week) and was found to be associated with a worsening of eating style. Television broadcasting and streaming platforms typically provide long and engaging material intended to capture the viewer’s attention within a dedicated time frame [40]. This format often stimulates a social aspect, as it is commonly shared with family and friends, and can become a modeling influence for children [41]. As a result, some frequently reported determinants of poor eating habits, including sedentary habits [39], mindless eating [11,42], and disruption of regular family meal planning [12], are likely to be found in association with consumption of TV/streaming content. Advertising might be another factor to consider, as food-related commercials could be enhanced by the sophisticated data analysis and tracking systems used by streaming services to deliver tailored advertisements to individual viewers [10], compared to traditional TV broadcasts [43].

A different interpretation should be sought for the associations between diet and time spent by children playing digital games (2.3 h/week). In previous research, digital games have been associated with reduced amounts of physical activity [44,45], reduced sleep duration and quality [46], and lower self-concept in children [47]. All these determinants, with their mutual influence, could potentially be seen as central factors in the association between digital games and adherence to the Mediterranean diet [47].

However, a gap still exists in existing research that has not been fully explored in the scientific literature. In fact, most previous studies on the association between digital media consumption and children’s health habits have focused primarily on overall screen time and television viewing. In contrast, the specific time spent using all other types of electronic devices, such as video game consoles, personal computers, and portable devices, has been addressed in only a limited number of studies [7,9,27,42]. Currently, neither the World Health Organization (WHO) nor the American Academy of Pediatrics (AAP) provide specific screen time limits for children over the age of six [48,49]. However, both organizations emphasize the importance of setting reasonable limits to screen time to promote the adoption of healthy habits by children [50,51].

As digital devices become more and more pervasive and begin to replace traditional means of performing everyday activities, such as doing homework or communicating with peers, parents need evidence-based information, specific for different digital devices, to promote healthier habits for future generations. Future research should delve deeper into these aspects to better understand the underlying mechanisms. Such investigations are essential to provide policymakers with robust evidence that can inform strategies and initiatives aimed at promoting healthy behaviors and improving dietary habits among children.

### Limitations

Some limitations of the present study should be acknowledged.

First, this study employs a cross-sectional design, which examines associations between variables at a single point in time but lacks the temporal data necessary to establish the sequence of events. As a result, the present study can only identify associations between potential determinants and adherence to the Mediterranean diet, but it cannot explore causal relationships.

Second, this study could not find an association between children’s diet and social media utilization. Moreover, social media use in our sample was reported to be close to zero, probably due to the young age of the sample. Therefore, these results may not be representative of other realities or of an older sample.

Third, this study used a self-administered questionnaire directed to mothers, which could be subject to various biases, including sampling bias, non-response bias, acquiescence bias, and social desirability bias. Children’s habits may also vary in different contexts (e.g., at home vs. at school), which mothers may not fully capture when responding to the survey.

Despite these limitations, this study is significant given the limited literature on this topic, providing new insights into the emerging role of various types of digital media consumption in influencing children’s adherence to the Mediterranean diet.

## 5. Conclusions

Activities such as playing digital games and watching TV or streaming platforms are significantly associated with poorer adherence to the Mediterranean diet among primary school children. These findings suggest that recommendations for promoting healthy eating habits in children should differentiate between types of digital activities when advising against prolonged media use. Further research is needed to explore this area and enhance our understanding of the factors linking children’s media use to their dietary habits, which could help shape more effective health promotion strategies.

## Figures and Tables

**Table 1:** Descriptive statistics of the sample.

Descriptive variable	Category	Frequency (%) N = 332
Sex ^a^	Male	161 (50.8%)
	Female	166 (49.2%)
Siblings ^b^	None	70 (21.2%)
	One or more	261 (78.9%)
Time dedicated to homework	No homework	9 (2.7%)
	<1 h	99 (29.8%)
	≥1 h	224 (67.5%)
Out-of-school activities ^c^	None	117 (35.6%)
	One or more	212 (64.4%)
Sports ^d^	None	55 (16.6%)
	One or more	276(83.4%)
Hours of sleep ^e^	Median (Q1–Q3)	9 (8–9)
Parent’s citizenship ^f^	Italian	255 (79.9%)
	Other	64 (20.1%)
Parent’s marital status ^g^	Married/cohabiting	282 (89.5%)
	Unmarried/divorced/widow	33 (10.5%)
Parent’s education ^h^	Middle school or less	45 (14.1%)
	High school diploma	137 (42.9%)
	University degree	137 (42.9%)
Family’s disposable income ^i^	Low	115 (35.8%)
	Medium	146 (45.5%)
	High	60 (18.7%)
Parent’s need for help in medical issues ^j^	Never/rarely	223 (70.8%)
	Occasionally/frequently/always	92 (29.2%)
Family members ^k^	≤3	88 (27.2%)
	4	77 (23.8%)
	>4	159 (49.1%)
Adherence to MD ^l^	Poor	45 (13.8%)
	Medium/high	281 (86.2%)
BMI ^m^	Underweight	13 (4.3%)
	Regular weight	199 (66.6%)
	Overweight	55 (18.4%)
	Obese	32 (10.7%)

Data not available in a 5 (1.5%), b 1 (0.3%), c 3 (0.9%), d 1 (0.3%), e 7 (2.1%), f 13 (3.9%), g 17 (5.1%), h 13 (3.9%), i 11 (3.3%), j 17 (5.1%), k 8 (2.4%), l 6 (1.8%), m 33 (9.9%).

**Table 2:** Distributions of the media usage score, time spent on digital games, and time spent watching TV or streaming platforms by KidMed groups.

		Adherence to the MD		*p*-Value
		Poor	Medium–High	
Time spent on social media	1st and 2nd tertiles	26 (12.4%)	184 (87.6%)	0.292
	3rd tertile	16 (17.8%)	74 (82.2%)	
Time spent on digital games	1st and 2nd tertiles	19 (9.1%)	190 (90.9%)	0.001
	3rd tertile	21 (23.6%)	68 (76.4%)	
Time spent on watching TV or streaming platforms	1st and 2nd tertiles	22 (10.1%)	197 (89.9%)	0.002
	3rd tertile	20 (25.0%)	60 (75.0%)	
Time spent on messaging	1st and 2nd tertiles	27 (13.4%)	174 (86.6%)	0.873
	3rd tertile	15 (14.9%)	86 (85.1%)	
Time spent on content creation	1st and 2nd tertiles	37 (14.2%)	223 (85.8%)	0.983
	3rd tertile	4 (16.7%)	20 (83.3%)	
Time spent on music	1st and 2nd tertiles	28 (13.3%)	183 (86.7%)	0.623
	3rd tertile	14 (16.3%)	72 (83.7%)	
Adult video games	1st and 2nd tertiles	31 (13.4%)	200 886.6%)	0.800
	3rd tertile	8 (16.0%)	42 (84.0%)	
Time spent on social media	1st and 2nd tertiles	35 (13.9%)	216 (86.1%)	0.899
	3rd tertile	5 (16.7%)	25 (83.3%)	
Time spent on phone or video calls	1st and 2nd tertiles	25 (12.2%)	180 (87.8%)	0.182
	3rd tertile	17 (18.9%)	73 (81.1%)	
Time spent on digital school assignments	1st and 2nd tertiles	28 (13.3%)	183 (86.7%)	0.614
	3rd tertile	13 (16.5%)	66 (83.5%)	
Time spent on web browsing in free time	1st and 2nd tertiles	30 (15.2%)	168 (84.8%)	0.666
	3rd tertile	12 (12.5%)	84 (87.5%)	
Time spent on watching video content	1st and 2nd tertiles	24 (11.8%)	180 (88.2%)	0.181
	3rd tertile	18 (18.2%)	81 (81.8%)	
Time spent on online gambling	1st and 2nd tertiles	40 (15.2%)	224 (84.8%)	0.339
	3rd tertile	1 (100.0%)	0 (0.0%)	
Time spent on other online activities	1st and 2nd tertiles	34 (14.0%)	209 (86.0%)	0.835
	3rd tertile	2 (22.2%)	7 (77.8%)	

**Table 3:** Backward stepwise of linear regression of KidMed score.

	Regression Coefficient	SE	*p* Value
Digital games (3rd vs. 1st and 2nd tertiles)	−0.64	0.29	0.028
Watching TV or streaming platforms (3rd vs. 1st and 2nd tertiles)	−0.66	0.29	0.024

Variables entered: sex, siblings, time dedicated to homework, out-of-school activities, sport, hours of sleep, parent’s citizenship, parent’s marital status, parent’s education, family’s disposable income, parent’s need for help in medical issues, family members, prosocial behavior, hyperactivity/inattention, peer relational problems, emotional symptoms, conduct problems, use of social media time.

## Data Availability

The datasets analyzed during the current study are not publicly available but are available from the corresponding author on reasonable request.
